# Gender stigma awareness is associated with adolescent risky health behaviors

**DOI:** 10.1371/journal.pone.0251332

**Published:** 2021-05-12

**Authors:** Karen Kwaning, Mitchell Wong, Kulwant Dosanjh, Christopher Biely, Rebecca Dudovitz

**Affiliations:** 1 David Geffen School of Medicine at UCLA, Los Angeles, California, United States of America; 2 UCLA General Internal Medicine and Health Services Research, Los Angeles, California, United States of America; 3 Department of Pediatrics, David Geffen School of Medicine at UCLA, Los Angeles, California, United States of America; Sun Yat-sen University, CHINA

## Abstract

**Objectives:**

Although racial stigma in school is associated with adolescent risky health behaviors, there are no studies investigating how gender stigma relates to adolescent risky health behaviors among low-income, minority youth. We sought to determine whether gender stigma awareness is associated with adolescent risky health behaviors (delinquency, fighting, and substance use) and whether this association is mediated by school disengagement (low perceived teacher support, low school engagement, cutting classes, and breaking school rules) among low-income, minority students.

**Methods:**

We analyzed cross-sectional survey data, collected from 2017 to 2019, from 412 high school students. Multi-level logistic regressions tested whether gender stigma awareness was associated with delinquency, fighting, and substance use, controlling for covariates, baseline behaviors, and clustering within schools. Mediation analyses tested whether school disengagement (low school engagement, perceived teacher support, cutting class, and breaking school rules) mediated these associations. Secondary analyses explored whether associations differed for male versus female, high-performing versus low-performing, and Latinx versus non-Latinx students.

**Results:**

In this predominantly Latinx (83%) sample, gender stigma awareness was associated with delinquency (AOR = 1.48, *P*< 0.001) and fighting (AOR = 1.15, *P*< 0.001). School engagement, perceived teacher support, breaking school rules, and cutting classes mediated 42.7% of the association between gender stigma awareness and delinquency and 65.42% of the association between gender stigma awareness and fighting. Gender stigma awareness was also associated with substance use for low-performing (AOR = 1.68, *P* = 0.003) and non-Latinx adolescents (AOR = 3.80, *P* = 0.03). School disengagement did not mediate the association between gender stigma awareness and substance use for non-Latinx students but mediated 50% of this association for low-performing students.

**Conclusions:**

Gender stigma awareness is associated with adolescent risky health behaviors. A decreased sense of acceptance in the school community and increased school misbehavior may mediate these associations. School environments that value and accept all students may better support adolescent health.

## Introduction

Adolescent risky health behaviors (delinquency, fighting, and substance use) exacerbate the school-to-prison pipeline and are associated with poor adult health outcomes: worse adult general health, depression, and suicidality [1]. Studies show that risky behaviors differ among racial/ethnic groups and genders. For example, recent studies show that Latinx adolescents had higher levels of substance use in early adolescence and African American students had higher final levels of smoking and marijuana use compared to students of other racial groups [2, 3]. Moreover, being an African American student, compared to being a white student, was associated with lower delinquent behavior scores, although adolescent fighting was most frequent among African American and Latinx students [4, 5]. Further, studies show that substance use dependence, delinquent behavior, and fighting is generally greater among males than females [6].

Critically, there are well-known associations between racial stigma and adolescent risky behaviors. For instance, feeling threatened by racial- or ethnic-based stereotypes is associated with poor academic performance, risky decision-making, and aggression [7]. Further, experiencing stigma based on racial stereotypes is associated with low academic performance in college, negative health outcomes in early adulthood (e.g., anxiety, hypertension, and inflammation), avoidance of healthcare, lack of communication with health care providers, and non-adherence to treatment plans in adulthood [8–11].

Other forms of stigma, such as stigma related to gender, may also impact adolescent health behaviors, particularly when considered in combination with racial and ethnic stigma. Gender stigma studies have largely focused on academic performance. For instance, sociological studies suggest that boys across all racial/ethnic backgrounds might feel the need to display masculinity through risky health behaviors, like fighting and delinquency, as opposed to through academic success [12, 13]. Although high school teachers may rate girls as more hardworking and less disruptive than boys and better engaging in quiet activities like reading, teachers may simultaneously endorse negative female stereotypes that include girls performing poorly at math [12, 14]. However, there are no studies that explore the association between gender stigma awareness and adolescent delinquency, fighting, and substance use among adolescents: a void in the literature that we endeavor to fill.

One strategy for assessing stigma is by measuring gender stigma awareness [15, 16]. Gender stigma awareness is the extent to which an adolescent is aware that the various judgements of their gender (e.g., boys are more delinquent than girls, boys are better at math; girls are more hardworking that boys, girls are not good at math) affect how people perceive and interact with them [17]. The examples listed are not a comprehensive list of positive and negative stigma that exist for adolescent boys and girls, but suggest stigma in general may have implications for adolescent risky behavior. Like other forms of stigma, gender stigma awareness may make students doubt that their teachers will judge their abilities fairly and support them academically. This theory is supported by previous studies showing that gender stigma is associated with poor academic performance, which is a known predictor of substance use [12, 18]. Consequently, students may feel a reduced sense of belonging in school communities [18].

We hypothesize that gender stigma awareness may result in school disengagement and increased rates of substance use, violence, and delinquency, particularly among racial and ethnic minority adolescents. Although studies suggest that adolescent risky health behaviors are greater among boys than girls, we hypothesized that gender stigma would negatively affect both boys and girls, given the mixed findings on the level of school disengagement along gender lines and based on the fact the associations between school disengagement and risky behaviors across both genders [19, 20]. However, we also hypothesized that associations between gender stigma awareness and health behaviors would be stronger for non-Latinx students than Latinx students, and likewise stronger for low-performing than high-performing students, as the intersections of these characteristics are also associated with negative behavior stereotypes.

Given the lack of studies examining the relationship between gender stigma and health behaviors for racial and ethnic minority youth, we tested whether gender stigma awareness was associated with risky health behaviors, and whether this association was mediated by school disengagement (low perceived teacher support, low school engagement, breaking school rules, and cutting classes). Further, because previous studies demonstrate differences in risky behavior among students with higher academic achievement [21] and among different racial/ethnic and gender groups [6], in secondary analyses, we investigated whether the relationship between gender stigma awareness and risky health behaviors was different for males versus females, high-performing versus low-performing students, and Latinx versus non-Latinx students. Ultimately, we further understand that the relationship between gender stigma awareness and health that may help identify potential leverage points for interventions to reduce disparities, similar to those used to mitigate racial stereotype threat—that is, feeling threatened when aware that one is being judged based on one’s race, which is associated with worse academic performance and health outcomes [20].

## Materials and methods

### Sampling and procedures

The study, including consent procedures, was approved by the University of California, Los Angeles, Institutional Review Board and by the participating school district (original approval date: 2017 23 February; study #:15–001190). We obtained written informed consent from all research participants. We conducted a secondary analysis of survey data from an evaluation of the Advancement Via Individual Determination (AVID) program in 5 large public high schools in a Californian urban school district. AVID is a college preparatory program targeting students in the academic middle (grade point average (GPA) of 2.0–3.5), who are from groups under-represented in higher education. For this study, 3 groups of students were recruited in 9^th^ grade, upon their transition to high school: 1) those eligible for and randomized to participate in the school’s AVID program; 2) those eligible for but not randomized to participate in AVID; and 3) students who were not eligible for AVID because their 8^th^ grade GPA was above 3.5. Students were recruited over two consecutive years from 2017 to 2018 and each completed a self-administered computerized baseline survey at their transition to high school (end of 8^th^ grade/beginning of 9^th^ grade). In addition, participants completed a follow-up survey in April or May of 2018 when one cohort was at the end of 9^th^ grade and the other was at the end of 10^th^ grade. Our outcome variable, gender stigma awareness, was only included in the follow-up survey, making our study cross-sectional. Notably, 412 of the 473 students who took the baseline survey also completed the follow-up survey, which represents 87% of the baseline sample. The majority of surveys were completed in school and alternative arrangements for survey completion were made for students who were absent on the day of administration. In such cases, surveys could be completed at a location in the community, like a library, or at the participant’s home if the participant and their parent requested it.

### Measures

#### Behavioral outcomes

We selected risky health outcomes that we hypothesized might be associated with gender stigma, particularly among minority adolescents, and may perpetuate the school-to-prison pipeline: delinquency, fighting, and substance use [12]. Measures of any delinquency, any fighting, and any substance use in the past year were based on the Youth Risk Behavior Survey, Monitoring the Future Survey, and the National Longitudinal Study of Adolescent to Adult Health [22, 23]. We asked students about their delinquent behavior in the past year, which was operationally defined as engaging in painting graffiti or signs on someone else’s property or in a public place, damaging someone else’s property, lying to a parent or guardian, stealing something from a store, running away from home, driving a car without its owner’s permission, entering a house or building to steal something, using or threatening to use a weapon to get something from someone, or selling marijuana or other drugs. We created a variable for any versus no delinquent behaviors in the past twelve months. In addition, students reporting any fighting in the past year that “did not involve their siblings” and “involved hitting, kicking, and pushing,” were considered to have engaged in any fighting. Further, students were asked about their substance use in the past year, which was operationally defined as smoking cigarettes, smoking an electronic vapor product, drinking alcohol other than a few sips for religious purposes, using marijuana, or using other illicit drugs (i.e., crack, cocaine, methamphetamines, prescription drugs, heroin, or inhalants). Students reporting any of these substance use behaviors in the past twelve months were considered positive for substance use.

#### Gender stigma awareness

We used the Gender Stigma Consciousness (GSC) subscale, a subscale from the Social Identities and Attitudes Scale that has been validated in adolescent populations [17, 24] to measure the awareness of gender stigma. For this measure, students are presented with five statements about gender and are asked to select the extent to which they agree with that statement ranging from “strongly disagree” to “strongly agree.” The five statements assess the extent to which an adolescent is aware that their gender affects how people perceive and interact with them regardless of whether the effect of their gender is perceived as positive or negative [17, 25]. The subscale does not refer to specific stigmatizing beliefs about gender and all items can be applied to male, female, and non-binary adolescents universally. An example from this subscale includes “my gender affects how people act towards me [17].” All items were placed by content validity experts (which included experts in stereotypes and gender and graduate students in educational psychology) and had content validity indices of 0.8 and above [17]. Each item is scored from 1 to 4 and then summed to create a scale that ranges from 5 to 20. Lower scores represent less gender stigma awareness. The Cronbach’s alpha for the subscale including all 5 items was 0.89, which is similar to other samples [17]. Gender stigma awareness was only measured at one time point, in the spring of 2018, when half the sample was in 9^th^ grade and half the sample was in 10^th^ grade.

#### School disengagement

Potential mediating measures of school disengagement included measures of teacher support, school engagement, skipping school, and breaking school rules.

Perceived teacher support was measured using items adapted from the Index of Parenting Style [26], which was designed to measure parent support. We replaced the words “parent” with “teacher.” Students were presented with seven statements about their teachers and other adults at school such as, “when I get a poor grade in school, my teachers encourage me to try harder” and “I feel supported to do my best in whatever I do by my teachers and other adults in my school.” Students were asked to select the extent to which they agreed with that statement, ranging from “none of the time” to “most of the time.” Each item was scored from 1 to 4 and then added to create a scale that ranges from 7 to 28. Lower scores represent low perceived teacher support. This scale had a Cronbach alpha of 0.89 in this sample.

School engagement was measured using the High School Survey of School Engagement [27]. Students were presented with twenty-nine items about their engagement in school, like “when I do well in school, it is because I work hard.” Students were then asked to select the extent to which they agreed with that statement, ranging from “strongly disagree” to “strongly agree.” Each item was scored from 1 to 4, added to create a scale that ranges from 29 to 116. Lower scores indicate low school engagement. The scale has a Cronbach alpha of 0.95 in this sample.

Finally, students indicated whether they “got in trouble for not following school rules” and “cut or skipped classes” in the past twelve months.

#### Covariates

All covariates were measured at baseline—meaning when the students were in eighth grade—and were selected for their potential to influence gender stigma awareness, delinquency, fighting, and substance use. They included gender, grade level, race/ethnicity, highest parental level of education, being born in the United States (U.S.), cohort, and whether the student was randomized to participate in AVID. Further, given that early engagement in adolescent risky behaviors is associated with a greater likelihood of engaging in those behaviors at a later age, we included students’ eighth grade delinquency, fighting, and substance use—which we designated as their baseline adolescent risky behaviors—as covariates [28].

### Statistical analysis

We standardized gender stigma awareness, perceived teacher support, and school engagement measures so that 1 unit corresponds to 1 standard deviation. We then conducted random-effects logistic regressions (students clustered within schools) testing whether gender stigma awareness was associated with each health outcome at follow-up, controlling for the relevant risky health behavior at baseline and covariates. As a secondary analysis, we tested whether the associations between gender stigma awareness and health behaviors varied by gender, race/ethnicity (Latinx versus non-Latinx), and low baseline grade point average (GPA <3.0) by stratifying our models based on gender, race/ethnicity, and GPA.

Next, we added potential mediators to each model to determine whether the associations between gender stigma awareness and each risky health behavior outcome diminished when controlling for school disengagement. We used the Karlson-Holm-Breen (KHB) method to determine the degree to which each school disengagement measure mediated the association between gender stigma awareness and risky adolescent health behaviors. The KHB method was developed to compare the estimated coefficients between nested non-linear probability models. It is a general decomposition method that estimates direct, indirect, and total effects of both continuous and discrete variables [29]. We used random-effects linear regressions for analyses of continuous outcomes and logistic regressions for analyses of dichotomous outcomes while accounting for clustering of students within schools. Robust standard errors to account for clustering within schools were applied. Missing data represented less than 1% for all variables. All analyses were done with Stata software, version 15 (StataCorp, College Station, Tex).

## Results

A total of 412 students completed both the baseline and follow-up surveys. The analytic sample consisted of these 412 students with data for both gender stigma awareness and at least one health outcome. Table 1 shows descriptive statistics from our follow-up data collection. As described in Table 1, the majority of students (83%) were Latinx and 39% of students were male. Just over half of students (54%) had at least one parent who graduated from high school. A majority of students (91%) were born in the United States. Consistent with the study design, nearly a third of students (28%) were randomized to participate in AVID. In this largely low-income minority sample, the mean gender stigma awareness score for male students was 8.1 while the mean gender stigma awareness score for female was 9.2 (*P* = 0.002). Both gender stigma awareness scores are nearly twice as high as the average gender stigma awareness score rating among ethnically diverse U.S. college students (4.7) [30].

**Table 1:** Demographics (N = 412).

Characteristic	N (%)/Mean (SD)
Male	162 (39.3%)
9^th^ grade	189 (45.9%)
10^th^ grade	223 (54.1%)
Race/Ethnicity	
White, non-Latinx	7 (1.70%)
Latinx	341 (82.8%)
Black or African American	10 (2.43%)
Asian or Pacific Islander	45 (10.9%)
American Indian or Native American	4 (0.97%)
Multiracial, non-Latinx	5 (1.21%)
At least 1 parent is a high school graduate	224 (54.4%)
Born in the U.S.	373 (90.5%)
8^th^ grade GPA	
3.0 or less	160 (38.8%)
3.1–3.5	105 (25.5%)
3.6–4.0	147 (35.7%)
Participated in AVID	116 (28.2%)
Mean Gender Stigma Awareness score: male	8.1 (3.4)
Mean Gender Stigma Awareness score: female	9.2 (3.8)
Baseline, Any Delinquency in past 12 months	156 (37.9%)
Follow-Up, Any Delinquency in past 12 months	224 (47.3%)
Baseline, Any Fighting in past 12 months	94 (22.9%)
Follow-Up, Any Fighting in past 12 months	83 (20.2%)
Baseline, Any Substance Use in the last 12 months	61 (14.8%)
Follow-Up, Any Substance Use in the last 12 months	71 (17.2%)
Mean School Engagement score	91.4 (14.4)
Breaking School Rules	129 (31.3%)
Cutting Classes	96 (23.3%)
Mean Perceived Teacher Support score	21.7 (4.6)

At baseline, 38% of students engaged in at least one delinquent behavior and 23% were in a physical fight in the past year, while 14.8% of students reported past year substance use. At follow up, 47.3% of the sample engaged in at least one delinquent behavior. We found that of the students who have engaged in at least one delinquent behavior, 40.6% of them reported having lied to a parent or guardian about where they had been or who they were with and 59.4% of students reported other delinquent behaviors. Further, 20% of students had been in a physical fight, and 17% engaged in substance use. The most common substance use reported at follow-up was alcohol (9.7%), followed by marijuana (9.5%), and smoking an electronic vapor product (8.3%). Fewer students reported using crack, cocaine, methamphetamines, prescription drugs, heroin, or inhalants (2.2%) or smoking traditional cigarettes (0.5%).

As described in Table 2, random-effects regression models revealed that gender stigma awareness was significantly associated with higher odds of delinquency (AOR = 1.48, 95% CI, 1.28–1.70) and fighting (AOR = 1.15, 95% CI, 1.06–1.23) after controlling for both participant characteristics and baseline health behaviors. Further, gender stigma awareness was not associated with substance use (AOR = 1.19, 95% CI, 0.97–1.47). However, the association between gender stigma awareness and substance use was significantly moderated by both race/ethnicity (interaction term *P* = 0.04) and academic performance (interaction term *P* = 0.01). As described in Table 3, stratified analyses demonstrated that the association between gender stigma awareness and substance use was larger for non-Latinx students (AOR = 3.80, 95% CI, 1.12–12.88) compared to Latinx students (AOR = 1.05, 95% CI, 0.83–1.33) and for low-performing students (AOR 1.68, 95% CI, 1.19–2.36) compared to high-performing students (AOR = 0.98, 95% CI, 0.78–1.22). Race/ethnicity and GPA did not moderate the associations between gender stigma awareness and delinquency or fighting and gender did not moderate associations between gender stigma awareness and any outcome.

**Table 2:** Associations between gender stigma awareness and adolescent health with and without adjusting for potential mediating school-related factors.

OUTCOMES	Model 1 (covariates only)			Model 2 (covariates + mediating factors)		
	AOR	95% CI	*P*	AOR	95% CI	*P*
Any delinquency	**1.48**	**1.28–1.70**	**<0.001**	**1.31**	**1.05–1. 64**	**0.02**
Any fighting	**1.15**	**1.06–1.23**	**<0.001**	1.05	0.53–2.07	0.90
Any substance use	1.19	0.97–1.47	0.10	0.96	0.52–1.77	0.89

AOR = adjusted odds ratio; CI = confidence interval.

Each model controlled for gender, cohort, race/ethnicity, highest parental level of education, whether the student was born in the U.S., grade point average, whether the students were randomized into AVID, and the applicable baseline health outcome.

Model 2 additionally controlled for potentially mediating school disengagement (school engagement, perceived teacher support, breaking school rules, and cutting classes).

**Table 3:** Associations between gender stigma awareness and substance use stratified by race and baseline academic achievement.

	**Non-Latinx**			**Latinx**		
	AOR	95% CI	*P*	AOR	95% CI	*P*
Any substance use	**3.80**	**1.12–12.88**	**0.03**	1.05	0.83–1.33	0.67
	**Low Achieving**			**High Achieving**		
	AOR	95% CI	*P*	AOR	95% CI	*P*
Any substance use	**1.68**	**1.19–2.36**	**0.003**	0.98	0.78–1.22	0.85

AOR = adjusted odds ratio; CI = confidence interval.

Each model controlled for gender, cohort, highest parental level of education, whether the student was born in the U.S., whether the students were randomized into AVID, and the applicable baseline health outcome. The race/ethnicity stratified models additionally controlled for baseline grade point average (GPA) and the achievement stratified models additionally controlled for race/ethnicity. Students with a baseline GPA of 3.0 or lower were considered Low Achieving. Students with a baseline GPA of 3.1 or higher were considered High Achieving.

As seen in Table 2, when mediators were added to regression models, associations between gender stigma awareness and all outcomes were substantially diminished. Results from our KHB models (Table 4) suggest that school engagement (10.18%), perceived teacher support (4.21%), breaking school rules (10.31%) and cutting classes (18%) significantly mediated the association between gender stigma awareness and delinquency; together, they mediated 42.7% of the association (*P* = 0.02). Collectively, the school disengagement factors mediated 65.42% of the association between gender stigma awareness and fighting (*P* = 0.02), largely driven by school engagement (25.93%) and breaking school rules (51.54%). School disengagement factors did not significantly mediate the association between gender stigma awareness and substance use among non-Latinx students (*P* = 0.062). However, school engagement (19.02%), perceived teacher support (5.50%), breaking school rules (11.41%), and cutting classes (13.67%) mediated the association between gender stigma awareness and substance use among low-GPA students. Together, these factors significantly mediated 49.6% of the association between gender stigma awareness and substance use for low-GPA students *(P =* 0.04).

**Table 4:** Estimated percent of associations between gender stigma awareness and risky health behaviors mediated by school disengagement.

Mediating Variable	Full Sample, Any Delinquency %	Full Sample, Any Fighting %	Non-Latinx, Any Substance use, %	Low-GPA, Any Substance use, %
School Engagement score	**10.18**	**25.93**	16.03	**19.02**
Perceived Teacher Support score	**4.21**	--*	9.62	**5.50**
Ever Breaking School Rules	**10.31**	**51.54**	10.43	**11.41**
Ever Cutting Classes	**17.99**	**0.66**	3.01	**13.67**
All	**42.67 (p = 0.02)**	**65.42 (p = 0.02)**	39.09 (p = 0.06)	**49.60 (p = 0.04)**

*No mediation effect.

Models controlled for gender, cohort, race/ethnicity, highest parental level of education, whether the student was born in the U.S., grade point average, and participation in AVID.

Note: P-value is for the indirect effect.

## Discussion

This is the first study, to our knowledge, to document associations between gender stigma awareness and adolescent risky health behaviors. We found that higher perceived gender stigma awareness was associated with increased odds of engaging in delinquency. Furthermore, fighting in a sample of mostly Latinx high school students in a large Californian urban city was associated with gender stigma awareness and there is increased odds of engaging in substance use among non-Latinx and low-GPA high school students in a Californian urban city. These are critical findings, as the risky health behaviors assessed here (i.e., delinquency, fighting, and substance use) can perpetuate the school-to-prison pipeline, and are associated with negative adult health outcomes [1, 16].

Additionally, the associations between gender stigma awareness and adolescent risky health behaviors were mediated by measures of school disengagement (school engagement, perceived teacher support, breaking school rules, and cutting classes), suggesting that gender stigma awareness may be one pathway through which schools can influence adolescent health. Creating school environments where students feel accepted, regardless of their gender or other personal characteristics, might help reduce risky health behaviors [31, 32].

The finding that associations between gender stigma awareness and substance use were stronger for non-Latinx students versus Latinx students and for low-GPA students versus higher-GPA students suggest that the experience of gender stigma awareness is intersectional and influenced by racial/ethnic and academic identity. Given that this sample is comprised largely of non-white students, our findings suggest that addressing factors that perpetuate gender stigma awareness may be particularly important for non-Latinx students of color and those who may have struggled academically.

Further, we did not observe significant differences in associations between gender stigma awareness and risky health behaviors for boys versus girls. Although we cannot determine the reason for this finding in the current analysis, we hypothesize that feeling stereotyped leads to negative feelings and school disengagement regardless of gender, both of which are risk factors for risky health behaviors, like delinquency, fighting, or substance use [33]. Furthermore, the gender difference in risky behaviors has diminished between boys and girls over the past two decades [3, 6, 34].

## Limitations

As with all research, there are limitations in our study. First, although we measured students’ gender stigma awareness, we did not include a measure of racial stigma. Likewise, because students were asked to identify as a male or female, we are unable to investigate whether adolescents who have a gender identity outside of the male-female binary have higher levels of gender stigma awareness. Therefore, although we cannot disentangle stigma based on gender versus other forms of stigma—and given that we found no gender differences in our associations in our sample—we suggest that any form of stigma can contribute to the likelihood that a student may engage in delinquency, fighting, or substance use.

Second, although we used the Gender Stigma Consciousness subscale, which is a validated subscale in the Social Identities and Attitudes Scale, the experience of stigma is intersectional and, thus, may be impacted by various parts of an adolescent’s identity, which may undermine the construct validity of our measure. Third, although we controlled for a variety of demographic characteristics and baseline academic achievement, we cannot rule out the possibility of unmeasured confounders. Our relatively small sample size limited our power and biased our results towards the null hypothesis. Also, our study largely relied on self-reported measures, which may be subject to social desirability and recall bias. Further, findings from our sample of mostly Latinx students from a single urban center may not be generalizable to other populations.

Finally, since our study is cross-sectional, it is impossible to determine the directionality of the relationship between gender stigma awareness and risky health behaviors. It is possible, for example, that engaging in delinquency, fighting, and substance use may expose adolescents to discrimination based on gender stigma, which may increase gender stigma awareness. Likewise, being less engaged in school may lead teachers to explicitly or implicitly endorse stigmatized beliefs of male and female students, which may increase an adolescent’s gender stigma awareness. Although the mediation analysis presented here is consistent with our hypothesis that gender stigma awareness leads to school disengagement and risky health behaviors, we cannot distinguish between potential alternative pathways.

## Conclusion

Despite these limitations, our findings have important implications for adolescent health. Namely, the experience of gender stigma awareness is associated with risky health behaviors among socioeconomically marginalized adolescents. Studies suggest that simple self-affirmation interventions can successfully mitigate the impact of race-based stereotype threat on academic performance and self-worth among children [35]. Future studies might investigate whether gender stigma awareness *causes* risky health behavior and school disengagement and whether similar self-affirmation strategies can be applied to gender stigma awareness to improve health outcomes. Future studies can consider investigating the associations between gender stigma awareness, school disengagement, and adolescent risky health behaviors in sexual and gender minority youth (i.e., adolescents who identify outside of the male-female gender binary). In addition, schools investing in strategies to increase school engagement and provide students with opportunities to develop supportive relationships with teachers and other school-related adults might improve students’ academic achievement and help to mitigate adolescent risky health behaviors. Although our current study focuses on gender stigma awareness in schools, adolescents may experience similar phenomena when interacting with other systems, including the healthcare system, and authority figures (such as physicians) [8]. Suitably, health care providers might consider their own implicit biases and acknowledge that gender stigma awareness exists when discussing the social determinants of adolescents’ health with patients and families. Lastly, child health advocates, pediatricians, and educational leaders can work together to improve adolescent health and reduce disparities by creating psychologically safe environments, where all adolescents feel seen and accepted.
