# Correction: Gender stigma awareness is associated with adolescent risky health behaviors

**DOI:** 10.1371/journal.pone.0274987

**Published:** 2022-09-16

**Authors:** Karen Kwaning, Mitchell Wong, Kulwant Dosanjh, Christopher Biely, Rebecca Dudovitz

There is an error in Table 1. The parameter “Participated in AVID” should have the N(%)/Mean SD values of “115 (27.9%)”, not “116 (28.2%)”. Please see the correct Table 1 here.

**Table 1:** Demographics (N = 412).

Characteristic	N (%)/Mean (SD)
Male	162 (39.3%)
9^th^ grade	189 (45.9%)
10^th^ grade	223 (54.1%)
Race/Ethnicity	
White, non-Latinx	7 (1.70%)
Latinx	341 (82.8%)
Black or African American	10 (2.43%)
Asian or Pacific Islander	45 (10.9%)
American Indian or Native American	4 (0.97%)
Multiracial, non-Latinx	5 (1.21%)
At least 1 parent is a high school graduate	224 (54.4%)
Born in the U.S.	373 (90.5%)
8^th^ grade GPA	
3.0 or less	160 (38.8%)
3.1–3.5	105 (25.5%)
3.6–4.0	147 (35.7%)
Participated in AVID	115 (27.9%)
Mean Gender Stigma Awareness score: male	8.1 (3.4)
Mean Gender Stigma Awareness score: female	9.2 (3.8)
Baseline, Any Delinquency in past 12 months	156 (37.9%)
Follow-Up, Any Delinquency in past 12 months	224 (47.3%)
Baseline, Any Fighting in past 12 months	94 (22.9%)
Follow-Up, Any Fighting in past 12 months	83 (20.2%)
Baseline, Any Substance Use in the last 12 months	61 (14.8%)
Follow-Up, Any Substance Use in the last 12 months	71 (17.2%)
Mean School Engagement score	91.4 (14.4)
Breaking School Rules	129 (31.3%)
Cutting Classes	96 (23.3%)
Mean Perceived Teacher Support score	21.7 (4.6)
